# Resilient Plant–Bird Interactions in a Volcanic Island Ecosystem: Pollination of Japanese Camellia Mediated by the Japanese White-Eye

**DOI:** 10.1371/journal.pone.0062696

**Published:** 2013-04-30

**Authors:** Harue Abe, Saneyoshi Ueno, Toshimori Takahashi, Yoshihiko Tsumura, Masami Hasegawa

**Affiliations:** 1 Field Center for Sustainable Agriculture and Forestry, Faculty of Agriculture, Niigata University, 94-2 Koda, Sado, Niigata, Japan; 2 Tree Genetics Laboratory, Department of Forest Genetics, Forestry and Forest Products Research Institute, Tsukuba, Ibaraki, Japan; 3 Satoyama Science Research Center, Faculty of Agriculture, Utsunomiya University, Utsunomiya, Tochigi, Japan; 4 Laboratory of Geographical Ecology, Toho University, Funabashi, Chiba, Japan; Institut Mediterrani d´Estudis Avançats (CSIC/UIB), Spain

## Abstract

Observations of interspecies interactions during volcanic activity provide important opportunities to study how organisms respond to environmental devastation. Japanese camellia (*Camellia japonica* L.) and its main avian pollinator, the Japanese White-eye (*Zosterops japonica*), offer an excellent example of such an interaction as key members of the biotic community on Miyake-jima, which erupted in 2000 and continues to emit volcanic gases. Both species exhibit higher resistance to volcanic damage than other species. We examined the effects of volcanic activity on this plant–pollinator system by estimating pollen flow and the genetic diversity of the next generation. The results showed that despite a decrease in *Camellia* flowers, the partitioning of allelic richness among mother-tree pollen pools and seeds decreased while the migration rate of pollen from outside the study plot and the pollen donor diversity within a fruit increased as the index of volcanic damage increased. In areas with low food (flower) density due to volcanic damage, *Z. japonica* ranged over larger areas to satisfy its energy needs rather than moving to areas with higher food density. Consequently, the genetic diversity of the seeds (the next plant generation) increased with the index of volcanic damage. The results were consistent with previously published data on the movement of *Z. japonica* based on radio tracking and the genetic diversity of *Camellia* pollen adhering to pollinators. Overall, our results indicated that compensation mechanisms ensured better pollination after volcanic disturbance.

## Introduction

The periods during any volcanic activity provide important opportunities to study how organisms respond to environmental devastation. Previous ecological studies on volcanic islands have mainly examined colonisation and primary succession of plant communities [1]–[6], but few studies have elucidated the recovery processes of late climax forest communities [7] with various symbiotic species interactions. Indeed, the distribution of the remaining organisms and their interactions hold the key to the subsequent biotic community in the successional process, for instance, by providing leaves, nectar, fruits, and seeds to diverse organisms. Therefore, we surveyed ecological processes and the consequences of several subsystems of biological communities on a volcanic island, Miyake-jima, Japan, which experienced an eruption in summer 2000.

To study an example maintenance mechanism in this community, we examined the effects of volcanic activity on pollen flow and reproduction of the common broad-leaved evergreen tree species, Japanese camellia (*Camellia japonica* L., Theaceae), mediated by pollinating birds. A small passerine bird, the Japanese White-eye (*Zosterops japonica*, Zosteropidae) is the main pollinator of *C. japonica* [8], [9]. On Miyake-jima, *C. japonica* and *Z. japonica* are the most important members of their temperate forest during natural recovery. Japanese camellia tolerates much more damage than other tree species [10]. The Forestry and Forest Products Research Institute [11] reported that *C. japonica* and *Eurya japonica* Thunb. (Theaceae) were the only species to germinate, especially in highly damaged forests. The avian population in general decreased greatly in response to reduced forest vegetation, but avian populations recovered throughout the winter once the emissions of volcanic ash and SO_2_ decreased; this was especially the case for *Z. japonica* and *Cettia diphone* (Cettidae) [12].

Abe & Hasegawa [9] (see 1–5, below) and Abe et al. [13] (6–7, below) previously reported the effects of volcanic activity on the reproductive success of *C. japonica*. 1) Volcanic gases negatively affected leaf survival and reduced flowering activity (proportion of flowering trees, number of flowers per flowering tree, flowering density) in heavily damaged areas. 2) Pollination rates were higher in heavily damaged areas than in less damaged ones. 3) Net fruit abortion rates were greater at heavily damaged sites than at less damaged sites; however early abortion rates tended to decrease with increasing volcanic damage. 4) The higher pollination rate compensated for fruit abortion in determining the final fruit- and seed-set rates in heavily damaged areas. Consequently fruit-set rates did not differ significantly among sites, and 5) seed-set rates tended to increase with increasing volcanic damage. 6) In areas affected by volcanic gases, the genetic diversity of pollen grains adhering to pollinator beaks was greater in areas with low flower density than in areas with higher flower density. 7) This result was consistent with bird pollinator movements tracked by radio [13]. These previous studies suggested that the genetic diversity of pollen pools delivered to maternal trees might greater in heavily damaged areas, but if strong spatial genetic structure exists within populations, efficient pollinator movement might still fail to benefit the genetic diversity of future generations. Therefore we examined the genetic diversity of pollen donors within a fruit to more directly evaluate the genetic diversity of the next generation.

In this study, we examined the effects of volcanism on pollen movement and on future plant generations by comparing the genetic diversity of seeds among sites with different levels of damage and flower densities caused by volcanic activity. The implications of these results for plant–pollinator system stability following environmental disturbances are discussed. We hypothesised that the resilient relationship between *C. japonica* and *Z. japonica* was important in maintaining the pollination system on this volcanic island, even in severely damaged areas, and lead to further bio-interactions.

## Materials and Methods

### Ethics Statement

We were given permission to access to Miyake-jima (in January, 2005) and its off-limits area (February–December, 2005) by the Miyake village office (license ID: 9934 for HA in January; based on a submitted research plan from February–December). No specific permissions were required for other locations and for the described field studies.

### Study Species

In eastern Asia, *Camellia japonica* (Theaceae) is a common broad-leaved evergreen tree species in climax forests [14]–[16]. *Camellia japonica* flowers from November to March, the local winter period, with a peak from January to February on Yaku-shima Island [17] and the Izu islands in Japan (Abe et al., unpublished data). This species is monoecious and produces conspicuous red bisexual flowers with a large quantity of nectar, which attracts birds because other foods (e.g., insects) provide insufficient nutrition in the winter. The number of flowers per flowering tree averaged 41.1±64.2 (SD), with a range of 2.7–191.5 on Miyake-jima in January, 2004 [9]. This is also self-incompatible [18]: flowers isolated from pollinating birds did not bear fruit [8]. Field observations revealed that the majority of animal visits on Miyake-jima and the adjacent Nii-jima Island [8], [9] were by *Z. japonica*, which appeared to be the primary pollinator. Kunitake et al. [8] used two types of exclosures to estimate the pollination effectiveness of *Camellia* flower visitors [19]. They demonstrated that 36.2% of open flowers (accessible to all visitors) produced fruits, whereas only 5.7% set fruit when caged to exclude larger animals but not insects. No bagged (self-pollinated) flowers fruited. Furthermore, the level of fruit set was saturated after only five visits by *Z. japonica* on Nii-jima [8]. The average number of seeds per fruit was 6.28±2.96 (SD; range, 4.37–12.7). Seed weight averaged 1.47±0.28 (SD; range, 1.09–1.89) on Miyake-jima in July 2005 [9]. The seeds are dispersed by rodents [20]. *Camellia japonica* L. var. *rusticana* (Honda), which is adapted to a heavy snowfall region facing the Japan Sea, flowers from April to May (spring) and is pollinated exclusively by small insects (Diptera, etc.) (Y. Watanabe, pers. comm. 2009).

### Study Areas

The Izu Islands, a volcanic group along the western rim of the Pacific Ocean, are characterised by a humid warm-temperate climate, with annual rainfall of more than 2,000 mm and an average air temperature of *c*. 17°C. The region is well known for the production of *Camellia* oil because a large number of *C. japonica* grow there. We chose as our study island the volcanically active Miyake-jima (55.44 km^2^ in area; N: 34.04.55°, E: 139.31.35°; altitude: 775.1 m:, located *c*. 180 km south of Tokyo), which erupted explosively during summer 2000. The volcano has approached quiescence, but small gas eruptions continue. The main vegetation before the eruption was an evergreen broad-leaved type dominated by *Castanopsis sieboldii* (Makino) Hatus., *Machilus thunbergii* Siebold & Zucc., and *C. japonica* [5], [6].

**Figure 1:**
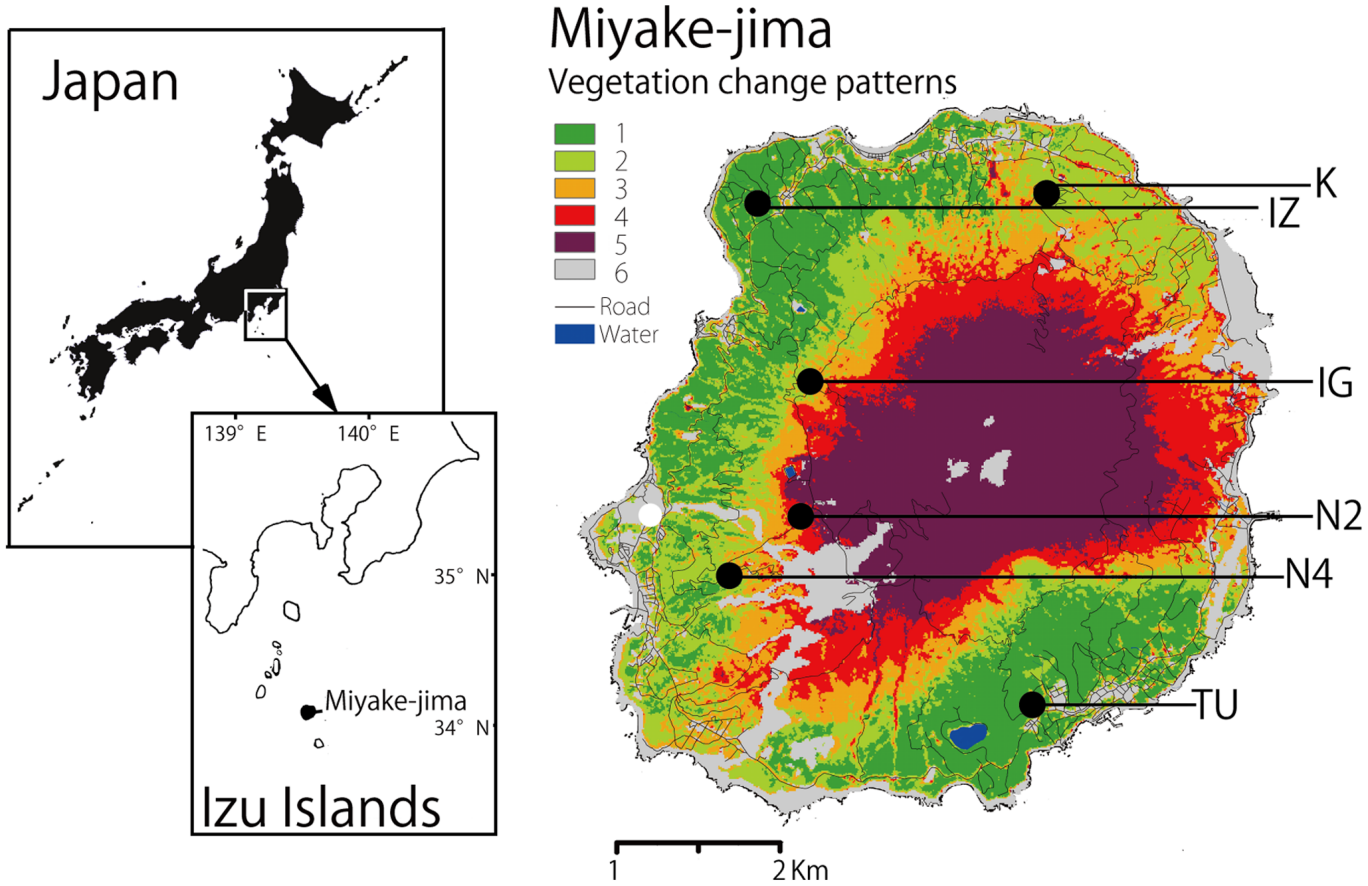
Study sites and patterns of vegetation. We established 0.3-ha plots at each of 6 study sites [Tubota (TU), Izu (IZ), Kamitsuki (K), Igaya (IG), Nanto-road 2 (N2), Nanto-road 4 (N4)] to assess how volcanic damage affected the ecosystem of Miyake-jima. Vegetation was categorized into six classes in order of increasing damage: 1, area covered by woody vegetation with no damage from the eruption; 2, areas in which woody vegetation was beginning to decline and be replaced by grassland; 3, area in which the forest had been reduced soon after the eruption, but grassland has begun to recover; 4, area in which the forest wood suffered heavy devastation following the eruption, and grassland is beginning to recover; 5, area in which the vegetation cover had been removed soon after the eruption and has not recovered; 6, poor vegetation area before the eruption (the area is residential or covered mainly by lava from the 1983 eruption) [21].

We established 0.3-ha plots (50×60 m) at each of six study sites [Tubota1 (TU1), Izu (IZ), Kamitsuki (K), Igaya (IG), Nanto-road2 (N2), and Nanto-road4 (N4)] to assess a variety of damage conditions caused by volcanic activities on Miyake-jima (Fig. 1).

### Index of Volcanic Damage

We used vegetation changes before and after the eruption to measure the volcanic damage to the island’s ecosystem. Takahashi et al. [21] evaluated the vegetation recovery process using satellite remote sensing and field vegetation surveys. Twelve multi-temporal Terra/ASTER and JERS-1/OPS satellite images were selected and combined into three time series datasets: 1) before the eruption, 2) within two years of the eruption, and 3) three or more years after the eruption. These datasets were analysed into seamless images of the maximum normalized difference vegetation index value. Based on the unsupervised classification of these three images, this island was divided into six vegetation change classes (1–6), which corresponded to the vegetation change patterns A–F in a previous study by Takahashi [21]: 1, woody vegetation not damaged by the eruption; 2, areas in which woody vegetation was beginning to decline and be replaced by grassland (woody plants were damaged but alive); 3, areas in which the forest was reduced soon after the eruption but grassland had begun to recover (some woody plants had died); 4, areas in which forests suffered heavy devastation following the eruption and grassland was beginning to recover (most woody plants died); 5, areas in which the vegetation cover was removed soon after the eruption and had not recovered; and 6, areas of poor vegetation before the eruption (residential or covered mainly by lava from a 1983 eruption). This index of volcanic damage (IVD) was assumed to increase from 1 to 6, but *Camellia* flowering trees did not exist at the sites with IVD 5 and 6. Therefore, we used four category sites with IVD 1 to 4.

### Flower Density

In winter 2004–2005, we established transects with the 0.3-ha plots next to roads at each of the six study sites. Each transect was 10 or 20 m wide (either 5 or 10 m along each side of a road) and 100–250 m long, and transects varied from 0.2–0.8 ha (Table 1). We recorded the number of flowers and estimated flower density (flowers ha^–1^) for each study site in January, 2005. We also counted the number of flowering trees within each 0.3-ha plot on each site.

**Table 1:** Summary information for the six *Camellia japonica* study sites on Miyake-jima, Japan.

Study site	IVD^1^	Census area (ha)	Flower density (ha^–1^)^2^	No. of flowering trees per plot^2^	No. of fruiting trees per plot	No. of mother trees sampled for fruit	No. of sampled fruits	No. of analysed fruits^3^	Mean no. of genotyped seeds per mother tree	Home range of *Zosterops japonica* 4
TU1 (Tubota1)	1	0.2	2544	48	48	10	50	25	19.8	0.26±0.24
IZ (Izu)	1	0.5	1777	37	32	9	45	31	21.87	–
K (Kamitsuki)	2	0.8	488	23	23	10	50	41	23.53	–
IG (Igaya)	3	0.8	998	26	20	9	45	35	27.59	1.97±1.20
N4 (Nanto-road4)	3	0.4	21	12	3	2	10	6	15.45	–
N2 (Nanto-road2)	4	0.3	28	17	8	8	40	27	22.56	–
Totals	–	3	5856	161	134	48	240	165	1068	–

1 Index of volcanic damage (IVD), which corresponds to the vegetation change patterns A–F in [19] in Figure 1; characterizes the degree of damage to forest vegetation caused by volcanic activity.

2 Flower density and no. of flowering trees in January, 2005 (flowering continued until March). Plots were 0.3-ha in size.

3 Analysed fruits were selected in which pollen donors were identified for more than four seeds by Cervus 3.0 [26] and seed samples were successfully amplified at more than five loci (average of 9.5).

4 Based on Abe et al. [13]. Values are means ± SD, in ha.

### Fruiting Tree Density, Sampling, DNA Extraction, and Genotyping

Because the distributions of *C. japonica* at the study sites tended to be clumped, at each site we selected a dense area of *C. japonica* trees as the study plot. In late July, 2005, we counted the number of fruiting trees in each 0.3-ha plot. We collected leaves from all mature (flowering) trees and five random fruits from each of two to ten mature trees standing at the centre of each plot. The proportion of trees sampled ranged from 20.8–100%, with an average of 36% (Table 1).

Genomic DNA of mature trees was extracted from dried or frozen leaves using a modified CTAB method [22]. Seed DNA was extracted from cotyledons by SDS–proteinase K treatment [23]: 1 g of cotyledons was crushed in a PCR tube with a toothpick, then 10 µL of reaction buffer [10 mM Tris–HCl (pH 8.3), 1.5 mM MgCl_2_, 50 mM KCl, 0.01% Proteinase K, 0.01% SDS] was added to the tube and incubated for 60 min at 65°C. The solution was diluted tenfold and used as DNA template for subsequent PCR.

Genotypes of all mature trees and a mean of 21.8 seeds per mother tree within the study plots were determined (Table 1) using 10 pairs of microsatellite PCR primers: MSE0030, MSE0045, MSE0049, MSE0051, MSE0053, MSE0062 and MSE0078 [24], MSCjaH38 [25], and MSCjaR02 and MSCjaQ11 [20]. We used seed samples that successfully amplified at more than five loci (average of 9.5). We also selected sample fruits to genetically analyse candidate pollen donors using Cervus 3.0 software [26] (see analysed fruits in Table 1); fruits in which we identified donors for more than four seeds were considered. Microsatellite amplification was then performed using Multiplex PCR Mix (Takara Bio, Shiga, Japan), according to the manufacturer’s protocol, in a PCR Thermal Cycler Dice Gradient TP600 (Takara Bio.). After an initial denaturation of 15 min at 95°C, 30 cycles were performed with denaturation for 30 s at 94°C, annealing for 90 s at 58°C, and extension for 90 s at 72°C, with final extension for 10 min at 72°C. The PCR amplification products were detected using an ABI Prism 3100 Genetic Analyzer (Applied Biosystems, Foster City, CA, USA). Individual genotypes were determined using Genotyper software (Applied Biosystems).

### Estimation of Pollen Movement

#### Genetic diversity of pollen pools

To estimate pollen movement, we calculated gene diversity, allelic richness, total paternity exclusion probability, and null allele frequency of the ten loci using Fstat 2.9.3 software [27] and Cervus 3.0 software [26]. When the genetic diversity of seeds from different mother trees is compared, the genetic composition of the mother tree can affect the genetic profile of the seeds. Therefore, to estimate the pollen pool of each mother tree, we excluded alleles derived from the mother trees based on the probability that alleles were transmitted via pollen, as in Fukue et al. [28].

Furthermore, by comparing genetic diversity among study sites, the partitioning of allelic richness among the mother tree pollen pools (*A*_st_) was calculated [29]. We used *A*_st_ rather than the index, *F_st_*, in this study because allelic diversity is more useful to estimate pollen movement. Because sample sizes of mature trees and seeds differed among the sites, we calculated allelic richness after the number of gene copies (*g*) was standardized to ten. We compared genetic diversity among sites using equation 1:
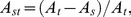
(1)where *A*_s_ and *A*_t_ indicate the mean allelic richness of subpopulations within populations and total allelic richness, respectively [29]. *A*_s_ was calculated as the average allelic richness of pollen pools within a mother tree across subpopulations minus one, because a subpopulation with a single allele would lack variation. *A*_t_ was calculated as total allelic richness of mature trees, assuming that established adult trees were potential pollen donors.

#### Use rate of flowering trees, pollen flow distance, migration rate of pollen, and pollen donor diversity

Paternity analysis of seeds was performed using Cervus 3.0 software. For each offspring tested, the paternity likelihood of each candidate pollen donor was calculated using a ratio of probabilities (the logarithm (base 10) of odds or LOD score) considering the genotypes of the offspring, its mother, and the candidate father as well as the population allele frequencies [30]. To evaluate the confidence that the most likely father (i.e., the one with the highest LOD score) was the true father, 10,000 simulations were performed in Cervus to estimate the critical *D* value–the difference between the highest and second highest LOD scores–at which the most likely father was the true father in 80% (relaxed confidence) or 95% (strict confidence) of cases. Both confidence levels gave similar results, so we only considered the relaxed confidence levels in subsequent analyses.

When pollen donors were identified, we calculated the number of pollen donors per flowering tree within each 0.3-ha plot to estimate the use rate of flowering trees as pollen donors by pollinators. The straight-line distance between a mother tree and its most likely father within the plot was considered the effective pollen dispersal distance. When we could not find a probable pollen donor within the plot, we assumed the pollen came from outside the plot. The migration rate was calculated as the number of pollen types from outside the plot divided by the total number of pollen types. The pollen donor diversity within a fruit was calculated as the number of detected pollen donors divided by the number of seeds for which candidate pollen donors were determined. For example, if there were six seeds in a fruit and pollen donors were identified for four of them, two pollen donors were outside the plot. Thus, the rate of unique pollen donors (pollen donor diversity) was 4/(6–2) = 1. If the pollen donors had the same genotype, we counted them as one pollen donor.

### Estimation of Genetic Diversity in the next Generation

To estimate genetic diversity in the next generation, we calculated gene diversity (expected heterozygosity), allelic richness, *F*_st_, and *A*_st_ using Fstat 2.9.3 [27] and Metapop 2.0 software [31].

### Statistical Analyses

To compare the correlation between the parameters of pollen movement (*A*_st_, pollen migration rate, and pollen donor diversity) and either IVD, flower density, or the interaction between IVD and flower density, we employed a generalized linear mixed model (GLMM) with the pollen movement parameters as the dependent variables. The values of the pollen movement parameters for one fruit or mother in a population may be correlated with the genetic diversity of other mothers in the population because the mothers may be genetically related and/or they may share pollinator guilds or environmental conditions (rain, temperature, etc.) Therefore we added the term population (site) as a random factor. Finally, a GLMM considered an identity link function in which the response followed a gamma distribution with lowest second-order Akaike information criterion. All statistical analyses were performed using R 2.15.0 (http://.r-project.org/), with the lme4 package for fitting mixed effects models.

## Results

### Flowering and Fruiting Tree Density, and Flower Density

Table 1 shows the numbers of flowering and fruiting trees within each 0.3-ha plot and the flower densities, which ranged from 21–2,544 flowers per hectare. The flower density was negatively correlated with IVD (Spearman’s *r* = –0.794, *P* = 0.05, *K* = 6, *N* = 6).

### Estimation of Pollen Movement

#### Genetic diversity of pollen pools

**Figure 2:**
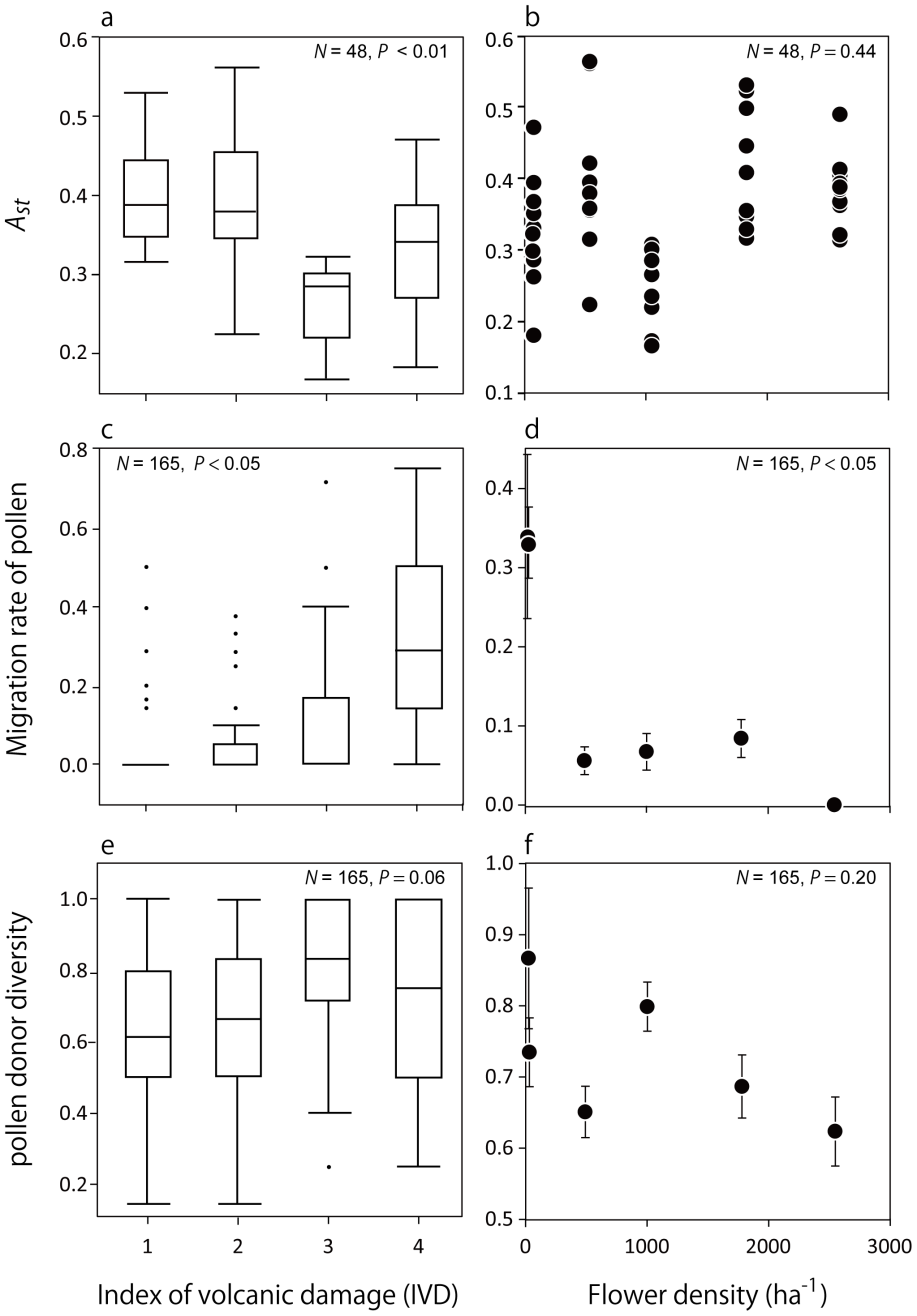
Effects of volcanic damage and *Camellia* flower density on *Camellia* pollen movement. A. Effects of the index of volcanic damage (IVD) on partitioning of allelic richness among a mother tree’s pollen pools (*A*_st_). **B,** Effects of flower density on *A*_st_. **C,** Effects of IVD on migration rate of pollen from outside a plot. **D,** Effects of flower density on migration rate of pollen from outside a plot. **E,** Effects of IVD on pollen donor diversity within a fruit. **F,** Effects of flower density on pollen donor diversity. In the box plots (**A, C, E**), the upper and lower bounds of and the horizontal line within each box represent the 75^th^, 25^th^, and 50^th^ percentiles, respectively; the whiskers range from the 90^th^ (above) to the 10^th^ (below) percentile. Dots indicate outliers. The whiskers extend from the ends of the box to the outermost data point that falls within the distances computed as follows: 1st quartile - 1.5*(interquartile range) 3rd quartile +1.5*(interquartile range) If the data points did not reach the computed ranges, the whiskers were determined by the maximum and minimum data points (excluding outliers). Closed circles and error bars (**D, F**) indicate mean and standard error. *P* value were determined using generalized linear mixed models (see text or Table S2).

The genotypes of all 161 mature trees and a total of 1,068 seeds (mean, 21.8 seeds per mother tree) from each of 2–10 mother trees per site were determined using ten microsatellite markers. The estimated null allele frequency ranged from –0.039 to 0.051 (Table S1), and at least one fragment per locus was observed, indicating no homozygotes for null alleles (Table S1). The cumulative paternal exclusion probability over 10 markers reached 0.9997. Genetic diversity and allelic richness of the mature trees and estimated pollen pools are provided (Table 2). The mean allelic richness was 4.297 for mature trees and 3.792 for estimated pollen. *A_st_* of the pollen pool ranged from 0.248–0.416 among the sites. Results of GLMM are shown in Table S2. *A_st_* was negatively correlated with IVD (*P<*0.01; Fig. 2a) and had no correlation with flower density (*P = *0.441; Fig. 2b). *A*_st_ was correlated with the interaction effects between IVD and flower density (*P*<0.0001).

**Table 2:** Genetic measures of diversity in six *Camellia japonica* plots on Miyake-jima, Japan.

	Expected heterozygosity		*F*_st_ of seeds^1,5^	Genetic diversity of pollen pools^2,5^	Allelic richness^3^			*A*_st_ 4		
Site	Mature trees	Seeds^5^			Mature trees	Seeds^5^	Pollen pools^5^	Seeds^5^	Pollen pools^5^	*P*ollen pools on *Z. japonica* beaks
TU1 (Tubota1)	0.696	0.572±0.043	0.174±0.005	0.641±0.128	4.267	3.816±0.191	3.631±0.211	0.300±0.009	0.383±0.047	0.35
IZ (Izu)	0.716	0.595±0.058	0.149±0.024	0.656±0.116	4.816	3.905±0.347	3.811±0.409	0.287±0.019	0.416±0.080	–
K (Kamitsuki)	0.670	0.580±0.030	0.113±0.017	0.640±0.113	4.007	3.807±0.233	3.699±0.459	0.241±0.019	0.395±0.098	–
IG (Igaya)	0.733	0.624±0.033	0.130±0.009	0.698±0.111	4.459	4.141±0.151	4.012±0.213	0.235±0.009	0.248±0.050	0.19
N4 (Nanto-road4)	0.707	0.583±0.036	0.136±0.009	0.634±0.121	4.083	3.724±0.111	3.817±0.070	0.217±0.032	0.310±0.012	–
N2 (Nanto-road2)	0.692	0.581±0.040	0.129±0.009	0.664±0.141	4.151	3.925±0.290	3.781±0.366	0.254±0.013	0.330±0.083	0.06

1 *F*_st_ is a measure of genetic differentiation among seeds within each mother tree.

2 Genetic diversity of pollen pools was calculated after removing the alleles of the mother trees.

3 Allelic richness of pollen pools was calculated after removing the alleles of the mother trees and standardizing the number of gene copies to 10.

4 *A*_st_ is the partitioning of allelic richness among populations (seeds or pollen pools) within each mother tree. *A*_st_ values of pollen pools on *Zosterops japonica* beaks were based on Table 5.4.b in Abe et al. [13].

5 Values are mean ± SD per mother tree.

#### Use rate of flowering trees, pollen flow distance, migration rate of pollen and pollen donor diversity

The use rate of flowering trees within 0.3-ha plots as pollen donors was the lowest at TU (37.5%) and the highest at IG (100%; Table 3). These rates tended to increase with the increasing IVD, except at N4 (because only two mother trees (sample size) were sampled for fruit; Table 1), which means pollinator birds were more likely to use flowering trees in more heavily damaged areas. The mean dispersal distance of pollen within the plots ranged from 10.52–18.71 m (Table 3). Results of the GLMMs are shown in Table S2. The migration rate ranged from 0–33.8% among sites (Table 3). It was positively correlated with IVD (*P*<0.05; Fig. 2c) and negatively correlated with flower density (*P*<0.05; Fig. 2d). There was also a correlation between the migration rate and the interaction effects of IVD and flower density (*P = *0.011). The pollen donor diversity within a fruit ranged from 0.62 to 0.96 among the sites and was positively correlated with IVD (*P* = 0.058; Fig. 2e). However, pollen donor diversity within a fruit was poorly correlated with flower density (*P* = 0.199; Fig. 2f) and with the interaction effects of IVD and flower density (*P = *0.534).

**Table 3:** Estimations of pollen movements in 0.3-ha plots on Miyake-jima, Japan.

Study site	Use rate of flowering trees within plot (%)	Pollen flow distance (m)	Migration rate of pollen into plots (%)	Pollen donor diversity (%)
TU1 (Tubota1)	37.5	13.99±4.49	0.0±0.00	62.4±24.2
IZ (Izu)	62.2	10.52±3.57	8.4±13.3	68.7±24.7
K (Kamitsuki)	82.6	15.95±9.98	5.6±11.2	65.1±23.1
IG (Igaya)	100.0	15.23±4.24	6.7±13.6	79.9±20.4
N4 (Nanto-road4)	75.0	18.71±5.81	33.8±21.5	86.7±8.9
N2 (Nanto-road2)	92.6	16.59±4.19	33.2±23.4	73.5±25.)

Pollen flow distance indicate mean straight-line distance from mother tree to estimated pollen donor, which was calculated for each mother tree (*N* = 48) (SD). Migration rate of pollen and pollen donor diversity were calculated for each fruit (*N* = 165) (SD). Pollen donor diversity indicate that number of pollen donors per number of seeds within a fruit.

### Genetic Diversity in the next Generation

The expected heterozygosity and allelic richness of the seeds are given in Table 2. The mean expected heterozygosity and allelic richness was 0.589 and 3.947, respectively. There were no differences in the genetic diversity of seeds among sites (Kruskal–Wallis test: *F* = 1.582, *P* = 0.122 in expected heterozygosity; *F* = 2.339, *P* = 0.074 in allelic richness, *K* = 6, *N* = 48; Table 2). *F*_st_ and *A_st_* ranged from 0.113–0.174 and 0.217–0.300, respectively, among sites and were positively correlated with each other (Spearman’s *r* = 0.738, *P*<0.001, *N* = 48). Results of the GLMMs are shown in Table S2. *A_st_* was negatively correlated with IVD (*P*<0.05) and positively correlated with flower density (*P*<0.01). *A*_st_ was also correlated with the interaction effects between IVD and flower density (*P*<0.005).

## Discussion

### Generality of the Efficient *Camellia* Pollination System

In this study, we hypothesised that the resilient relationship between *C. japonica* and *Z. japonica* was important in maintaining the pollination system on this volcanic island. The *A*_st_ values among mother-tree pollen pools decreased while the migration rate of pollen from outside the study plot and pollen donor diversity increased with IVD and also tended to increase as the *Camellia* flower density decreased (Fig. 2). These results corresponded to an enhanced efficiency in pollination system and support our hypothesis. In areas with low food density due to volcanic damage, *Z. japonica* might range over larger areas to satisfy its energy needs rather than moving to areas with higher food (flower) density. Is this interaction unusual on volcanic islands or more commonly seen in on other areas?

Japanese White-eyes are typical omnivores that from spring to fall may eat many types of insects, spiders, fruits, and seeds [32]. However, in winter in temperate forests, the availability of fruits and insects is limited, and the birds feed primarily on *C. japonica* floral resources, which can comprise 70–90% of their winter diet on the Izu Islands (Y. Kunitake and M. Hasegawa, unpubl. data, 1999) [8]. In New Zealand, Murphy and Kelly [33] quantified food available to passerines in a *Nothofagus* forest at Craigieburn, where fruit and insects were also scarce in winter. Total invertebrate energy was only about twice as high in summer as in winter, a smaller difference than expected, which might indicate that some birds use flower nectars more than insects. Many *Z. japonica* also migrate from the main Honshu Island of Japan to the Izu Islands in winter [32] by hopping along the several islands between them. Our previous study indicates a large change in *Z. japonica* densities accompanying changes in *C. japonica* flower densities [9]. Thus, *C. japonica* nectar serves as the most important food resource for resident White-eyes as well as White-eyes from the main island [8]. *Camellia japonica* benefits from this relationship by being pollinated. Thus, this relatively specialised winter-time pollination system may be general in other areas.

### Effects of Large Volcanic Disturbance to Facilitate the Pollination System

In contrast, the large volcanic disturbance appears to have facilitated this relationship more strongly compared with normal conditions. The pollination system persisted even after flower density decreased. This persistence was also observed in areas with low *Camellia* flower density caused by insect damage on the adjacent Nii-jima Island [34]. However the White-eye has greater effects on pollination on Miyake-jima than on Nii-jima; the indirect effects of the bird pollination system on fruit set rate were larger on Miyake under the volcanic activity [35]. Based on the GLMM analysis (Fig. 2; Table S2), pollen flow was more correlated with IVD and/or the two-factor interaction between IVD and flower density than with flowering density alone. Thus, both the direct effects of volcanic damage on the birds (i.e., they cannot remain in one place because volcanic damage harm the birds themselves, physically) and the indirect effects (food shortage via low flower density) affected pollen movement. The data from the IG site (IVD 3, moderate flower density) suggested how the indirect and direct factors affected pollen movement. This site tended to have higher pollen-pool genetic diversity (*A*_st_ and pollen donor diversity) than the N4 (IVD 3, but lower flower density) or K (IVD 2, lower flower density) sites (Table 2). Radio tracked White-eyes stayed more than one week at IG, although they did not stay at N2 [13] or N4 (H. Abe, pers. observe.). Even though the index of volcanic damage was higher at IG (it suffered dense gases, scoria, and ash deposits in 2000), volcanic gases subsequently flowed north-westerly away from the area and towards N2 and N4 (see Fig. 1), so flower density was gradually recovering and *Zosterops* could remain for a relatively long time at IG [9]. As a result, the birds could use flowering trees evenly within lager-sized home ranges at IG (1.97 ha) than TU (IVD 1, higher flower density, home range size 0.26 ha; Table 1), which led to higher use of flowering trees as pollen donors within the IG plot than TU, more pollen donor diversity, and lower *A*_st_ of pollen pools and migration rates at IG than N2 and N4. The volcanic damage on Miyake-Jima directly affected the pollinator bird movements and overall food abundance (invertebrate and fruit density as well as *Camellia* flowers), which might explain why *Zosterops* were so much more dependent on *Camellia* flowers, which lead to more efficient pollination on the volcanic island than under normal conditions.

### Resistance of the Animal-pollination System against Natural Disturbance

Generally, decreased plant (and flower) density caused by a large-scale natural disaster negatively affects pollination systems (e.g. [36], [37]). For example, in the case of the ornithophilous flowering plant *Pavonia bahamensis* (Malvaceae), hurricanes can reduce both available resources and the pollination rate, decreasing fruiting rates [36]. However, recent studies have demonstrated the presence of resistance mechanisms by which plants deal with such disturbances and/or low population densities [38]–[48], as seen in this study. Do these systems adapt to natural disturbances by same mechanism as in our study?

Ward et al. [46] reviewed that pollen dispersal is widespread among low-density tropical trees, ranging from a mean of 200 m to over 19 km for species pollinated by small insects or bats. Bronstein and Hossaert-McKey [47] reported that even though a hurricane decimated fig trees and fig wasps, asynchronous flower maturation and long-distance wasp dispersal allowed for a quick recovery of reproduction. Plant rarity and pollination by small insects are expected to cause low outcrossing rates and nearest-neighbour mating [49]. Counter to this expectation, Ward et al. [46] found that neotropical trees typically outcross and are self-incompatible, with long-distance pollen dispersal that relies on animal-mediated pollination. In the present study, pollen flow due to *Z. japonica* was also enhanced at the low flower densities associated with volcanic damage [9], [13], as demonstrated that the *A*_st_ of pollen pools tended to be lower (*P* = 0.4) and the migration rate of pollen within a fruit was higher (*P* = 0.01) in areas with lower flower density (Fig. 2).

Birds are highly mobility, which might ensure long distance dispersal under low food density. Herrera [50] reported that widely dispersed but attractive food resources could allow the expansion of the home ranges of individual birds. In this way, birds may act as mobile links connecting distant plant habitats [51]. Open terrestrial landscapes may enable this behaviour in migratory animals, but the dispersal effect depends on the scale of the disturbance and the species involved [52]. *Zosterops* are generally resilient and found in severely disturbed landscapes. *Zosterops japonica*, a migratory bird [32], uses distant plant habitats with IVD 3–4 during volcanic activities [9], [13]. *Zosterops japonica* has become a very important pollen vector in disturbed landscapes in Hawaii [53], [54] as is *Z. lateralis* in New Zealand [55]. *Zosterops* may function as a mobile link in not only our system but also in other systems.

The resistance of the animal-pollination system to disturbance can be ascribed to four properties: (1) an attractive food resource coupled with a shortage of food elsewhere in the habitat [50]; (2) the presence of highly mobile animal vectors that can traverse long distances (e.g. fig wasps, bees, and birds [46], [48], [52]); (3) plant self-incompatibility [46]; and (4) the strength of the interdependence between the animal vector and its plant partner. Thus, in our study, the presence of highly mobile *Z. japonica* combined with the strength of the relationship between the self-incompatible *C. japonica* and *Z. japonica* facilitated the maintenance of the pollination systems on this volcanic island.

### Effects of Pollination System on the Genetic Diversity of Future Generations

Natural disturbances have can promote plant reproduction and recruitment [56]. In our previous study [9], the maternal reproductive efficiency (e.g., seed set rate) of *C. japonica* increased in areas that were heavily damaged by volcanic activity. However, if strong spatial genetic structure exists within the study plot, efficient pollinator movement might still fail to benefit the genetic diversity of seeds. In this study, we also revealed that this system under natural disturbance facilitated paternal reproductive success (the genetic diversity of seeds) as well as maternal reproductive success [9]; the *A*_st_ values of seeds were lower in large damaged areas with low flower densities (*P*<0.05; Table S2). Ueno et al. [57] reported that Moran’s *I* spatial autocorrelation coefficient of *C. japonica* revealed weak genetic structure within a 4-ha permanent plot located on Tsushima Island, between the Japanese Archipelago and the Korean Peninsula. The *F*_st_ value of mature trees was 0.041 in this study, which also indicated week genetic structure. Higher genetic diversity of seeds might stem largely from higher genetic diversity of pollen through bird pollinator movements (Table 2, 3 and S2; Fig. 2) in areas with weak spatial genetic structure.

When discussing the effect on genetic diversity, we must factor in population (flower density) size reduction. Populations that have experienced a recent reduction in effective population size might suffer reduced allele numbers and heterozygosity at polymorphic loci; importantly, the loss of alleles occurs faster than the loss of heterozygosity (e.g. [58]). If there is no significant association between *F*_st_ and IVD, *F*_st_ might respond slower than *A*_st_ to disturbance. In this study, the *A*_st_ and *F*_st_ of seeds were strongly correlated. These values increased as IVD and/or flower density decreased (Table S2), which means the volcanic activity did not affect genetic diversity of the next generation, boding well for both conservation and evolutionary adaptation.

We must also consider the effects of abortion by *Camellia* itself. We collected the seed samples after early abortion, which tended to decrease as the IVD increased [9]. Indeed, the pollen donor diversity contributing to seed production did not differ significantly among sites (Fig. 2, Table S2) although the genetic diversity of pollen pools on bird beaks was higher at more damaged sites [13] (Table 2). This finding might suggest that abortion effects existed at less damaged sites, but the mechanism is not known.

Overall, these results indicated that compensation mechanisms ensured better genetic diversity in the next generation of *C. japonica* on Miyeke-jima by the large scale natural disturbance of volcanic activity. Therefore, we should not plant trees more than necessary to restore volcanically devastated sites. Appropriate forest management should be taken to control risks of genetic disturbance by human activity.

### Future Research

Different species interact in complex antagonistic (e.g., predator–prey) or mutualistic (e.g., pollinator–plant) networks [59]. Using a simple dynamic model, Bascompte et al. [60], [61] showed that the coevolutionary network patterns may facilitate species persistence; mutualistic networks can thus be regarded as the architecture of biodiversity. Therefore, both physiological tolerance in a species and ecological tolerance to changed interactions after volcanic disturbance may be key factors in restoring the island ecosystem (e.g. [47]). Thus, one interaction might generate other interactions. For example, the pollinating *Z. japonica* birds might act as mobile links, dispersing other kinds of seeds from relatively undamaged areas to highly damaged areas, promoting forest recovery on this island. To guide future ecosystem management in changing environments, we should understand how remaining mutualistic networks under huge disturbances stabilize these networks, which promote forest recovery.

## Supporting Information

Table S1 **Characteristics of the 10 microsatellite loci in** ***Camellia japonica*** **based on 161 flowering trees.** *A,* Number of alleles; *H_o,_* Observed heterozygosity; *H_e,_* Expected heterozygosity; Null, Null allele frequency (estimated); Excl, Paternity exclusion probability.
(DOC)

Table S2 **Results of Generalized Linear Mixed Modelling with a population included as random effect evaluating the effect of different volcanic damage (IVC) and flower density on pollen movements and next genetic diversity (seeds).** *A*_st_ indicates the partitioning of allelic richness among populations (seeds or pollen pools) within each mother tree. Chi-square values are the results of likelihood ratio test (anova) tests. Figures in bold indicate significant effects (*P*<0.05). Pollen donor diversity indicates that number of pollen donors within 0.3-ha per number of seeds within a fruit.
(DOC)
