# Supplementary material for: Resilient Plant–Bird Interactions in a Volcanic Island Ecosystem: Pollination of Japanese Camellia Mediated by the Japanese White-Eye
Source: PLoS One. 2013 Apr 30;8(4):e62696. doi: 10.1371/journal.pone.0062696 (PMC3639980; doi:10.1371/journal.pone.0062696)
Supplement: Table S1 — Characteristics of the 10 microsatellite loci in Camellia japonica based on 161 flowering trees. A, Number of alleles; Ho, Observed heterozygosity; He, Expected heterozygosity; Null, Null allele frequency (estimated); Excl, Paternity exclusion probability. (DOC) [file pone.0062696.s001.doc]

**Table S1** Characteristics of the 10 microsatellite loci in *Camellia japonica* based on 161 flowering trees.

| Locus | *A* | *H_o_* | *H_e_* | Null | Excl. | Accession No. |
| --- | --- | --- | --- | --- | --- | --- |
| MSE0030 | 10 | 0.718 | 0.796 | 0.048 | 0.612 | AB461369 |
| MSE0045 | 13 | 0.725 | 0.803 | 0.051 | 0.619 | AB461368 |
| MSE0049 | 3 | 0.625 | 0.587 | –0.039 | 0.313 | AB361047 |
| MSE0051 | 16 | 0.786 | 0.852 | 0.038 | 0.700 | AB461365 |
| MSE0053 | 8 | 0.527 | 0.547 | 0.023 | 0.321 | AB461367 |
| MSE0062 | 13 | 0.868 | 0.887 | 0.008 | 0.766 | AB461364 |
| MSE0078 | 14 | 0.714 | 0.771 | 0.039 | 0.591 | AB461366 |
| MSCjaH38 | 13 | 0.679 | 0.725 | 0.032 | 0.535 | AB016190 |
| MSCjaQ11 | 14 | 0.763 | 0.759 | –0.009 | 0.589 | AB211361 |
| MSCjaR02 | 9 | 0.568 | 0.566 | 0.005 | 0.316 | AB211362 |
| Mean | 11.3 | 0.697 | 0.729 | 0.020 | 0.536 |  |

*A,* Number of alleles; *H_o_,* Observed heterozygosity; *H_e_,* Expected heterozygosity; Null, Null allele frequency (estimated); Excl, Paternity exclusion probability.
