# Supplementary material for: Resilient Plant–Bird Interactions in a Volcanic Island Ecosystem: Pollination of Japanese Camellia Mediated by the Japanese White-Eye
Source: PLoS One. 2013 Apr 30;8(4):e62696. doi: 10.1371/journal.pone.0062696 (PMC3639980; doi:10.1371/journal.pone.0062696)
Supplement: Table S2 — Results of Generalized Linear Mixed Modelling with a population included as random effect evaluating the effect of different volcanic damage (IVC) and flower density on pollen movements and next genetic diversity (seeds). A st indicates the partitioning of allelic richness among populations (seeds or pollen pools) within each mother tree. Chi-square values are the results of likelihood ratio test (anova) tests. Figures in bold indicate significant effects (P<0.05). Pollen donor diversity indicates that number of pollen donors within 0.3-ha per number of seeds within a fruit. (DOC) [file pone.0062696.s002.doc]

**Table S2** Results of Generalized Linear Mixed Modelling with a population included as random effect evaluating the effect of different volcanic damage (IVC) and flower density on pollen movements and next genetic diversity (seeds).

|  | Sample size | IVD | | |  | Flower density | | |  | IVD × Flower density | | |
| --- | --- | --- | --- | --- | --- | --- | --- | --- | --- | --- | --- | --- |
|  |  | *d.f.* | *X*^2^ | *P* |  | *d.f.* | *X*^2^ | *P* |  | *d.f.* | *X*^2^ | *P* |
| *A*_st_ of pollen pools | 48 | 1 | 7.3228 | **0.007** |  | 1 | 0.5935 | 0.441 |  | 3 | 19.519 | **< 0.001** |
| *A*_st_ of seeds | 48 | 1 | 4.1333 | **0.042** |  | 1 | 7.8665 | **0.005** |  | 3 | 13.632 | **0.004** |
| Migration rate of pollen to 0.3-ha site | 165 | 1 | 5.1699 | **0.023** |  | 1 | 5.2621 | **0.022** |  | 3 | 11.117 | **0.011** |
| Pollen donor diversity | 165 | 1 | 3.5908 | 0.058 |  | 1 | 1.6527 | 0.199 |  | 3 | 2.1898 | 0.534 |

*A*_st_ indicates the partitioning of allelic richness among populations (seeds or pollen pools) within each mother tree. Chi-square values are the results of likelihood ratio test (anova) tests. Figures in bold indicate significant effects (*P* < 0.05). Pollen donor diversity indicates that number of pollen donors within 0.3-ha per number of seeds within a fruit.
